# Factors affecting recurrence in sigmoid volvulus

**DOI:** 10.12669/pjms.39.1.6882

**Published:** 2023

**Authors:** Ercan Korkut, Rifat Peksoz, Esra Disci, Sabri Selcuk Atamanalp

**Affiliations:** 1 Ercan Korkut MD, Assistant Professor, Department of General Surgery, Faculty of Medicine, Ataturk University, Erzurum, Turkey; 2 Rifat Peksoz MD, Assistant Professor, Department of General Surgery, Faculty of Medicine, Ataturk University, Erzurum, Turkey; 3 Esra Disci MD, Assistant Professor, Department of General Surgery, Faculty of Medicine, Ataturk University, Erzurum, Turkey; 4 Sabri Selcuk Atamanalp MD, Professor, Department of General Surgery, Faculty of Medicine, Ataturk University, Erzurum, Turkey

**Keywords:** Sigmoid volvulus, Recurrence, Volvulus attack, Predisposing factor

## Abstract

**Objectives:**

Recurrence is a relatively common outcome following endoscopic decompression in sigmoid volvulus (SV). This study aims to evaluate the factors affecting recurrence in SV.

**Methods:**

In 434 patients with SV treated between June 1986 and January 2022, probable recurrence-affecting factors including age, age of SV onset, gender, dietary habit, defecation habit, altitude of living area, and SV attack count were analyzed in prospectively collected data.

**Results:**

Of 434 patients, 111 (25.6%) had recurrent SV with mean 1.6 ± 2.3 of volvulus episodes (range: 1-21 attacks). SV recurrence demonstrated a significant linear increase with age (14.3%, 17.1%, 21.5%, and 29.9%, respectively, in young, middle aged, mature, and elderly patients, p < 0.001). Recurrent SV was also significantly higher in male gender (28.5% vs 12.7%, p = 0.004), high-fiber diet habit (32.9% vs 17.7%, p < 0.001), and living at high altitude (26.9% vs 12.5%, p = 0.047). Although SV recurrence was higher in patients with chronic constipation, the difference was not significant (36.7% vs. 20.3%, p = 0.594). When compared with that of the patients with mature onset or elderly onset patients, SV attack count was significantly higher in young-onset cases (1.3 ± 0.9 and 1.1 ± 0.3 vs 4.6 ± 6.9, p < 0.001).

**Conclusion:**

Elderliness, early-onset, maleness, high-fiber diet habit, high altitude, and most likely chronic constipation may be the practical clinical parameters of recurrent SV. These parameters, as well as the presence of previous SV episode history, must be considered in the decision-making process in the elective treatment of SV.

## INTRODUCTION

Sigmoid volvulus (SV), the wrapping of the sigmoid colon around its mesentery causing closed-loop colonic obstruction, tends to recur in mean 25% (between 0 - 85%) of cases, which principally depends on the treatment method and is associated with high mortality and morbidity.^1^,^2^ Although old age, male gender, high-fiber dietary habit, chronic constipation, and living at high altitude are known as predisposing factors for primary SV,^3^,^4^ their causative role in recurrent SV, as well as the role of age of onset and presence of previous volvulus history, are not identified in the literature to date.^5^ In an attempt to evaluate the current roles of the above-mentioned factors in recurrence development in SV, we analyzed the largest single-center SV series over the world^6^ including 1,046 cases treated in our clinic.

## METHODS

Among 1,046 patients with SV treated over 55.5 years between June 1966 and January 2022, 434 cases (41.5%), whose data were collected prospectively after June 1986, were evaluated. From the point of age, a total of 434 patients was divided into four groups; patients ≤ 19 years old were included in the ‘young patient group’, while those from 20-39 years old in the ‘midlife patient group’, 40-59 years old in the ‘mature patients group’, and ≥ 60 years old in the ‘elderly patient group’. According to gender, dietary habit, defecation habit, and altitude of living area, patients were included into two groups in each; male patients in ‘male group’ and female patients in ‘female group’; patients with high-fiber diet in ‘high-fiber diet group’ and others in ‘normal diet group’; patients with chronic constipation are in ‘constipation group’ and others in ‘normal defecation group’, patients who live ≥ 5.000 feet in ‘high altitude group’ and < 5.000 feet in ‘normal altitude group’. Finally, 111 recurrent cases were divided into four groups according to the age of SV onset; the age of the first volvulus attack was ≤ 19 years in the ‘young-onset group’, 20-39 years in the ‘midlife onset group’, 40-59 years in the ‘mature onset group’, and ≥ 60 years in the ‘elderly onset group’. For each patient, age, gender, dietary habit, defecation habit, altitude of living area, and the previous volvulus history (the age of the first volvulus attack and the total volvulus attack count) were noted.

Patients with early recurrence (during the hospitalization period) were treated by emergency surgery, while elective surgery was recommended in some selected patients with recurrence anamnesis, and the receivers were treated surgically.

In statistical analysis, data were expressed as mean ± standard deviation for the numerical variables, while as number and percentage for categorical variables. The distribution of the data was examined with histogram graphics. Ensemble averages were compared with Student’s t-test in normally distributed variables, while the Mann-Whitney U test was preferred in non-normally distributed variables. Categorical data were compared with the chi-square test. Pearson correlation analysis was also used in the evaluation of the numerical variables. One way Anova and Tukey from post hoc tests were used in data analysis. Data were analyzed at a 95% confidence interval and statistical significance was set at p < 0.05. Statistical analysis was performed by using SPPS version 22.0 software (IBM Corporation, Armonk, New York, United States).

This study was approved by the institutional review board (Ethical Committee of Ataturk University Faculty of Medicine, 27.01.2022, B.30.2.ATA.0.01.00/88). Written informed consent was obtained from all participants.

## RESULTS

In prospectively evaluated 434-case SV series, the mean age was 60.4 ± 15.9 years (range: 7-98 years). Of the patients, 111 (25.6%) had recurrent SV anamnesis with a mean 1.6 ± 2.3 of volvulus episodes (range: 1-21 attacks) and 19 patients (17.1%) had multiple attacks when the last attack was excluded. The previous treatment option was endoscopic decompression in 109 patients (98.2%) except for two cases (1.8%) with surgical decompression alone.

As demonstrated in Table-I, of 434 recurrent SV cases, seven (1.6%) were in the young patient group, 35 (8.1%) in the midlife patient group, 158 (36.4%) in the mature patient group, and 234 (53.9%) in the elderly patient group. SV recurrence was present in 14.3% of the patients in the young patient group, whereas this ratio was 17.1%, 21.5%, and 29.9%, respectively, in the midlife patient, mature patient group, and elderly patient groups. The mean age was 57.7 ± 15.2 years in nonrecurrent patients (range: 7-98 years), whereas it was 68.2 ± 15.3 years (range: 17-91 years) in recurrent cases. In statistical evaluation, the recurrence of SV demonstrated a significant linear increase with age (p < 0.001). Additionally, SV recurrence was statistically higher in the male group than that of the female group (28.5% vs. 12.7%, p = 0.004); in the high-fiber diet group than that of the normal diet group (32.9% vs. 17.7%, p < 0.001); and in the high altitude group than that of the normal altitude group (26.9% vs. 12.5%, p = 0.047). Although SV recurrence was higher in the constipation group than that of the normal defecation group, the difference was not statistically significant (36.7% vs. 20.3%, p = 0.594).

**Table-I:** Data in total 434 patients and statistical analysis.

Group/Parameter	Total	Recurrence	Statistical analysis
Young patient group	7 (1.6%)	1 (14.3%)	Independent Sample T test p < 0.001
Midlife patient group	35 (8.1%)	6 (17.1%)	
Mature patient group	158 (36.4%)	34 (21.5%)	
Elderly patient group	234 (53.9%)	70 (29.9%)	
Male group	355 (81.8%)	101 (28.5%)	Pearson Chi-square test p = 0.004
Female group	79 (18.2%)	10 (12.7%)	
High-fiber diet group	225 (51.8%)	74 (32.9%)	Pearson Chi-square test p < 0.001
Normal diet group	209 (48.2%)	37 (17.7%)	
Constipation group	139 (32.0)	51 (36.7%)	Pearson Chi-square test p = 0.594
Normal defecation group	295 (68.0)	60 (20.3%)	
High altitude group	394 (90.8%)	106 (26.9%)	Pearson Chi-Square test p = 0.047
Normal altitude group	40 (9.2%)	5 (12.5%)	

As demonstrated in Table-II, of 111 recurrent SV cases, eight (7.2%) were in the young-onset group, whereas 14 (12.6%) in the midlife onset group, 33 (29.7%) in the mature onset group, and 56 (50.5%) in the elderly onset group. In these groupings, the mean age of onset was 55.3 ± 18.1 years (range: 7-89 years). Of the patients, 101 (91.0%) were male. Although male patient rates were statistically higher than that of the female patient rates in all groups, there was no significant difference between the groups (p = 0.154). The mean attack number was 1.6 ± 2.3 (range: 1-21 attacks), whereas it was 4.6 ± 6.9, 2.7 ± 2.8, 1.3 ± 0.9, and 1.1 ± 0.3, respectively, in the young-onset, midlife onset, mature onset, and elderly onset groups. In statistical analysis, when compared with that of the mature onset and elderly onset groups, the total volvulus attack number was significantly higher in the young-onset group (p < 0.001). Similarly, the multiple attack ratio was 17.1% in general, whereas it was 62.5%, 57.1%, 12.1%, and 3.6%, respectively, in the young-onset, midlife onset, mature onset, and elderly onset groups. The multiple attack rate statistically demonstrated a significant linear decrease with advancing age of onset (p < 0.001).

**Table-II:** Data in recurrent 111 cases and statistical analysis.

Parameter/Group	Young onset group	Midlife onset group	Mature onset group	Elderly onset group	Total	Statistical analysis
n	8 (7.2%)	14 (12.6%)	33 (29.7%)	56 (50.5%)	111	-
Age (mean, range)	54.4 (17-98)	58.8 (20-97)	64.7 (41-88)	74.6 (60-91)	68.2 (11-98)	-
Age of onset (mean±SD, range)	15.0±5.1 (7-19)	32.3±5.6 (20-39)	51.8±5.4 (40-59)	68.8±7.7 (60-89)	55.3±18.1 (7-89)	-
Gender (male – female)	7/1 (87.5%/12.5%)	12/2 (85.7%/14.3%)	30/3 (90.9%/9.1%)	52/4 (92.9%/7.1%)	101/10 (91.0%/9.0%)	Mann-Whitney U test p = 0.154*
Total volvulus attack (mean±SD, range)	4.6±6.9 (1-21)	2.7±2.8 (1-11)	1.3±0.9 (1-5)	1.1±0.3 (1-3)	1.6±2.3 (1-21)	One way Anova test Pearson correlation test p < 0.001**
Multiple attack (mean)	5 (62.5%)	8 (57.1%)	4 (12.1%)	2 (3.6%)	19 (17.1%)	Chi-square test p < 0.001

* Male patients-female patients in all groups: significant, between all groups: non-significant.

** Young onset-mature onset groups: significant, young onset-elderly onset groups: significant, between others: non-significant.

In this series, 11 of 189 females (5.8%) were pregnant, one (9.1%) with SV recurrence. Similarly, one (12.5%) of 8 patients (7.6%) with Parkinson’s disease demonstrated recurrent SV. On the other hand, two patients (0.2%) with Hirschsprung’s disease had recurrence anamnesis.

## DISCUSSION

Dolichosigmoid, a dilated and elongated sigmoid colon with a long mesentery, is the main anatomical prerequisite in the development of SV.^3^,^7^-^9^ This anatomical variant is rarely congenital, which clarifies childhood SV, whereas it is generally acquired, that is principally affected by age, gender, dietary or defecation habits, environmental factors such as altitude, and some disorders including Hirschsprung’s or Chagas disease.^3^,^7^-^11^ Likely that Dolichosigmoid may also be effective in the development of recurrent SV, particularly in patients with decompression alone.^9^,^12^

Advanced age is a predisposing factor in the primary SV, additionally, early SV onset in childhood or teenage associated with elderliness is a practical indicator for recurrent SV.^4^,^6^,^13^ It is believed that SV incidence builds up over time due to the increasing Dolichosigmoid rate.^14^ For this reason, recurrent episodes may reach up to 86% in the elderly population.^15^ On the other hand, although SV is uncommon in childhood and adolescence due to the rarity of congenital dolichosigmoid,^3^,^7^,^16^ recurrent SV is not a surprising outcome in adults and elders with early SV onset.^13^,^17^ In our series, the rarity of both the primary and the recurrent SV in the young and middle-aged patients together with the linear increase with age in addition to the excess of the recurrent SV particularly in young-onset elders, supports this idea.

In males, the shape of sigmoid colon mesentery is relatively different and Dolichosigmoid is more common. For this reason, SV is seen about four times more in males.^7^-^9^,^18^ Male gender is also an indicator for recurrent SV,^4^ as was demonstrated in our series. Undigested fiber arising from high-fiber diet habits causes bulky fecal material and fecal loading in the colon.^19^ Similarly, chronic constipation due to bed defecation habits prolongs the colonic transit period.^3^ All of these increase intracolonic pressure and worsen the colonic elastogenesis resulting in Dolichosigmoid and related SV.^3^,^10^ However, the cause and effect relation between chronic constipation and Dolichosigmoid is more complex than thought. Following its development, Dolichosigmoid causes chronic constipation in itself.^9^ By any means, more than half of SV patients declare a high-fiber diet habit, while one third of them are chronically constipated people in endemic SV regions.^8^,^10^ The results of our study revealed a similar tendency to the recurrent SV in patients with high-fiber diet habits and most likely in those with chronic constipation.

In high altitudes, lower atmospheric pressure causes the expansion of intracolonic gases including carbon dioxide, methane, and hydrogen, as a consequence, chronic increased intracolonic pressure worsens the elastogenic stricture of the colon wall causing Dolichosigmoid and related SV.^11^ For this reason, SV is common in high-altitude regions such as Bolivia, the Andes, Peru, India, and Turkey.^6^,^20^-^22^ Similarly, the recurrent SV rate is reported to be between 7% and 45% in such countries.^20^,^22^ A similar predisposition was also found out about SV recurrence in the present study.

On the other hand, previous SV episode history is one of the most known risk factors in the development of SV recurrence.^12^ The high rates of our cases with previous SV attacks as well as multiple episodes demonstrate the predisposing role of the previous disease arising from no definitive treatment in recurrent SV.

Finally, some rare physiological or pathological entities that are effective in the development of the primary SV, may also trigger the recurrence in SV. Among these, pregnancy may cause recurrent SV by preventing the physiological untwisting of the sigmoid colon arising from the narrowed intraabdominal volume.^23^ Hirschsprung’s disease may also be associated with a high SV recurrence rate due to the presence of an aganglionic colonic segment, even if in patients with sigmoid colectomy.^24^ Similarly, Parkinson’s disease may cause a neuronal loss in the myenteric plexus in addition to its destructive effects on the spinal cord, by this way, it may activate the recurrence in SV.^25^

## CONCLUSIONS

Conclusively, although the rarity of the patients in some subgroups and the long duration of the evaluation period are the limitations of this study, some factors including elderliness, early-onset, maleness, high-fiber diet habit, living at high altitudes, and most likely chronic constipation seem as causative factors in recurrent SV. Although assessment of the general health situation by a rating system such as the American Society of Anesthesiologists physical status classification score is the principal consideration in decision making process in elective surgical sigmoid colectomy in SV, as the practical clinical parameters of SV recurrence, the above-mentioned parameters as well as the presence of previous SV episode history must also be considered.

### Authors’ Contribution:

**EK SSA:** Data collection, manuscript writing, revision of the final draft. **RP ED:** Data collection, revision of the final draft. **SSA:** is responsible and accountable for the accuracy and integrity of the work.
